# Repaglinide-loaded solid lipid nanoparticles: effect of using different surfactants/stabilizers on physicochemical properties of nanoparticles

**DOI:** 10.1186/s40199-015-0128-3

**Published:** 2015-09-21

**Authors:** Hossein Ali Ebrahimi, Yousef Javadzadeh, Mehrdad Hamidi, Mohammad Barzegar Jalali

**Affiliations:** Biotechnology Research Center, Tabriz University of Medical Sciences, Tabriz, Iran; Department of Pharmaceutics, Faculty of Pharmacy, Tabriz University of Medical Sciences, Tabriz, Iran; Zanjan Pharmaceutical Nanotechnology Research Center (ZPNRC), Zanjan University of Medical Sciences, Zanjan, Iran; Department of Pharmaceutics, School of Pharmacy, Zanjan University of Medical Sciences, Zanjan, Iran

## Abstract

**Background:**

Repaglinide is an efficient anti-diabetic drug which is prescribed widely as multi-dosage oral daily regimens. Due to the low compliance inherent to each multi-dosage regimen, development of prolonged-release formulations could enhance the overall drug efficacy in patient populations.

**Methods:**

Repaglinide-loaded solid lipid nanoparticles (SLNs) were developed and characterized in vitro. Various surfactants were used in this study during the nanocarrier preparation procedure and their corresponding effects on some physicochemical properties of SLNs such as size, zeta potential; drug loading parameters and drug release profiles was investigated. Stearic acid and glyceryl mono stearate (GMS) were used as lipid phase and phosphatidylcholin, Tween80, Pluronic F127, poly vinyl alcohol (PVA) and polyvinyl pyrrolidone (PVP) were used as surfactant/stabilizer.

**Results:**

The results showed some variations between formulations; where the Tween80-based SLNs showed smallest size, the phosphatidylcholin-based SLNs indicated most prolonged drug release time and the highest loading capacity. SEM images of these formulations showed morphological variations and also confirmed the nanoscale size of these particles. The FTIR and DSC results demonstrated no interaction between drug and excipients. The *invitro* release profiles of different formulations were studied and observed slow release of drug from all formulations. However significant differences were found among them in terms of their initial burst release as well as the whole drug release profile. From fitting these data to various statistical models, the Peppas model was proposed as the best model to describe the statistical indices and, therefore, mechanism of drug release.

**Conclusion:**

The results of this study confirmed the effect of surfactant type on SLNs physicochemical properties such as morphological features, loading parameters, particle sizes and drug release kinetic. With respect to the outcome data, the mixture of phosphatidylcholin/Pluronic F127 was selected as the best surfactant/stabilizer to coat the lipid core comprising stearic acid and GMS.

## Introduction

Diabetes mellitus is a wide spread chronic disease with serious life-threatening effects on different parts/functions throughout the body. The majority of diabetic patients are found to be type ІІ diabetes mellitus, i.e., (non-insulin-dependent). To manage this type of diabetes, different classes of oral anti-diabetic drugs are used routinely in the market [1]. Repaglinide, a drug in the class of meglitinides, is a new anti-diabetic agent which approved by FDA as an oral hypoglycemic drug in 1998. It reduces the fasting glucose concentration by stimulating the release of insulin from pancreatic beta cells. Repaglinide differs from other anti-diabetic drugs in its structure, binding profile, duration of action and mode of excretion. It is practically insoluble in water, while capable of being rapidly absorbed from GI tract after oral administration: a typical behavior in class II Biopharmaceutical Classification System (BCS II) drugs. Repaglinide undergoes extensive first pass metabolism in liver which, collectively, results in poor oral bioavailability of the drug (56 %). It has a high plasma protein binding (more than 98 %) and its half-life in systemic blood circulation is about 1 h. Repaglinide causes hypoglycemia following oral administration, although the extent of this feature is less than the sulphonylureas [2]. These properties have proposed the drug as a good candidate for sustain oral delivery formulations. In this way, many attempts have been made as application of different carriers such as liquisolid compacts [3], gastroretentive floating delivery system [4] and different polymeric nanoparticles [5, 6].

In the recent decades, solid lipid nanoparticles (SLNs) have attracted many attentions as nanotechnology-based drug delivery systems. Their potential advantages such as the possibility of controlled drug release and targeting, feasible incorporation of lipophilic as well as hydrophilic drugs, remarkable biocompatibility and low biotoxicity, capability of avoiding organic solvents in production cycle and ease of scale up, have provoked many research projects exploiting the possible use of these nanostructures in drug delivery. Oral administration of SLNs can potentially increase the lymphatic transport of drugs, thereby causing decreased first pass hepatic metabolism and increased oral bioavailability of the drug [7].

The two main ingredients of SLN-based formulations are lipid and surfactant/stabilizer. Surfactants reduce the interfacial tension between the hydrophobic surface of lipid core and the aqueous environment and, therefore, stabilize the SLNs structure. The effect of surfactant on the properties of SLNs has been investigated and described in several studies. Drug-independent properties of SLNs such as particle size, stability and zeta potential were studied by majority of these researches [8–20] and, to the best of our knowledge, drug-dependent properties such as drug incorporation and drug release have not yet been evaluated as a function of surfactant/stabilizer composition [12, 18]. Such study will potentially help to develop efficient carriers to deliver drug by SLNs. In addition it may shed a light on the mechanism(s) by which the physicochemical features of a drug may interact with the stabilizer in formation of a drug-loaded SLN. So in this study it was tried to investigate the effects of different surfactants on some important attributes of a typical BCS II drug like repaglinide to be formulated as a SLN-loaded nanopharmaceutical.

## Materials and methods

### Materials

Repaglinide was a gift from Alborz Darou Pharmaceuticals, (Qazvin, Iran); pluronic F127 was purchased from Sigma, USA. Stearic acid (Art No. 8.00673.1000, Merck, Germany), glyceryl mono stearate (GMS, MYVEROL™, KERRY, Denmark), phosphatidylcholin (Art No. E80-103, Lipoid, USA), tween80 (Art No. 601809, Titrachem, Iran), poly vinyl pyrrolidone (PVP; 30 KDa, Rahavard Tamin, Iran) and poly vinyl alcohol (PVA; 145 KDa, Art No. 1.41353.1000, Merck, Germany) were purchased and applied. Other ingredients and solvents were from HPLC or chemical laboratory purity grades, as needed, and were purchased locally.

### Preparation of SLNs

SLNs were prepared by a modified solvent diffusion method, where ethanolic phase containing 150 mg stearic acid, 50 mg GMS and 50 mg repaglinide was diffused in aqueous phase under magnetic stirring condition (1000 g) at 50 °C with an addition rate of 1 ml/min and, then, the resulting dispersion was allowed cooling until the room temperature. Surfactants were added to alcoholic or aqueous phases according to their solubility. After formation, SLNs were centrifuged at 50,000 g and lyophilized. Phosphatidylcholin (100 mg), poly vinyl pyrrolidone (PVP) (1 %), Tween 80 (1 %), pluronicF127 (100 mg), poly vinyl alcohol (PVA) and a mixture of phosphatidylcholin/Tween80 (100 mg/ 1 %) and phosphatidylcholin/pluronicF127 (100 mg/100 mg) were used as stabilizer during the preparation procedure.

### Particle size and zeta potential analysis

Particle size and zeta potential distribution of the prepared SLNs population were determined based on dynamic light scattering (DLS) technique using a laser scattering particle size/zeta analyzer (Malvern, model Nano ZS, UK). For particle size analysis, SLNs were diluted in water until their refractive index (RI) became to 1.4.

### Repaglinide assay

High performance liquid chromatography (HPLC) analysis was applied for determination of repaglinide concentrations in samples from drug loading or in vitro drug release studies. A HPLC system (Waters, model 1515, USA) was equipped with a UV C_18_ reverse phased column (MZ analytical, 250*4.6 mm, Mainz, Germany) and the mobile phase consisted of acetonitrile: phosphate buffer, 10 mM (pH = 2.5) at a ratio of 4:1. Chromatography was performed at flow rate of 1 ml/min at room temperature and the samples absorbance was determined at 245 nm by diode array detector (Waters, dual absorbances, model 2487, USA).

### Determination of drug loading and entrapment efficiency

For this purpose, an accurately weighed amount of freeze dried of repaglinide-loaded SLNs was dissolved in ethanol and the quantity of the drug was determined spectrophotometrically at wavelength of 245 nm. The drug loading efficiency (DLE) was computed as below:

$$ Drug\  loading\  Efficiency=\frac{amount\  of\  drug*100}{total\  weight\  of\ SLN} $$

To calculate the entrapment efficiency (EE), the SLNs dispersion were centrifuged at 50,000 g for 45 min and, then, the supernatant phase was decanted and the amount of drug in this phase was determined by ultraviolet spectroscopy at 245 nm. The entrapment efficiency was computed by the equation:

$$ Entrapment\  efficiency=\frac{amount\  of\  drug*100}{amount\  of\  drug\  used\  in\  formulation} $$

### Morphology studies by scanning electron microscopy

The size, shape and morphological properties of the prepared nanocarriers were studied by scanning electron microscopy (SEM). Lyophilized nanoparticles were coated by gold and at acceleration voltage of 7 kV were observed by SEM (MIRA TESCAN, Czech Republic).

### Fourier transform infrared (FTIR) spectroscopy

The freeze dried samples were homogenously mixed with potassium bromide and the mixture was then compressed into discs by a hydraulic compressor through applying pressure of about 10 tones in 2 min. The discs were placed in infrared light pass and the infrared spectrum was recorded in region of 400–4000 cm^−1^using a FTIR spectroscope (Bruker, Tensor 27, Germany).

### Differential scanning calorimetry

Differential scanning calorimetry (DSC) was carried out on the lyophilized samples of the nanocarriers using a differential scanning calorimeter (Mettler-Toledo Inc., STARe system, US). 2 mg of accurately weighed lyophilized samples were placed on the aluminum pan and sealed. An empty aluminum pan was used as standard. Both pans were heated from 25 °C to 300 °C at a scanning rate of 10 °C per minute under nitrogen flow. The quantity of heat flow was determined as a function of samples temperature and it was used to study the thermal behaviors of nanoparticles.

### In vitro drug release profile study

The in vitro drug release test was carried out thrice using dialysis bag diffusion technique. Dialysis bags (cut-off = 12 KDa) were immersed in distilled water for 24 h before use. 3 ml of SLNs dispersion equivalent to 2 mg repaglinide was placed into the dialysis bag, sealed by double-folding on both sides, and immersed in 100 ml of phosphate buffered saline (PBS) at 37 ° C. The system was placed on continuous magnetic stirring at 50 rpm. At predetermined time intervals, 1 ml sample was taken from receiver phase and the amount of repaglinide was analyzed by HPLC method as described above. The volume of release media was kept constant by addition of rectified volumes of PBS after each sampling stage. The sink conditions were preserved at whole time of the test in such a way that the repaglinide concentration in receiver phase remained less than 15 % of its saturation concentration in the same medium. Concentration of samples was determined by HPLC method as mentioned above and the release profile of each formulation was plotted graphically as percent drug released against the time in a bi-numerical mode.

### Drug release kinetic estimation

In order to determine the best model to describe the kinetic parameters of the drug release profiles, the experimental release data were fitted to multiple statistical models. The corresponding equations of each model were given in Table 1. The linear form of each equation (*y* = *at* + *b*) was fitted to the pure or transformed data (according to each equation) and the squared correlation coefficient (r^2^) between time and y parameter (related to release data based on each equation) was determined in each case. The r^2^ was used as a critical parameter to evaluate strength of correlation in models and also to compare each model with others. In addition, the mean relative error (MRE) was used as another critical parameter in order to overcome possible biases of r^2^ [21]. MRE was calculated as follows:

**Table 1:** Applied statistical models to release data of repaglinide

Function	Equation
Zero order	*M*_0_ − *M* = *kt*
First order	*lnM*_0_ − *lnM* = *kt*
Higuchi	*M*_0_ − *M* = *kt* ^0.5^
Peppas	*M*_0_ − *M* = *kt* ^*n*^
Hixon-crowell	*M*_0_^1/3^ − *M* ^1/3^ = *kt*
Square root	*M*_0_^1/2^ − *M* ^1/2^ = *kt*
weibull	${M}_0-M=1-{e}^{-{\left(\frac{t}{T_d}\right)}^{\beta }}$
Linear wagner	*Z*_0_ − *Z* = *kt*
Log wagner	*Z*_0_ − *Z* = *klnt*

*M*_*0*_and *M* are the remained amounts of the drug in carrier in the time zero and t respectively. Z is the probit of surface of nanoparticles along time. K is the equation constant and β is a shape factor of the equation figure

$$ MRE=\left(M-C\right)*100/C $$

Where C is the experimental data and M is the calculated release data from the applied model.

### Statistical analysis

All experiments and measurements were replicated thrice. The mean diameter and standard deviations were calculated by Microsoft Excel 2007. The effect of different surfactants on the release profiles properties and kinetics were compared using a one-way analysis of variance using SPSS 16. In this analysis, statistical difference was significant when *p* < 0.05.

## Results and discussions

### Preparation of solid lipid nanoparticles

The solvent diffusion method was applied in the current study to prepare SLNs. This method is based on the rapid diffusion of an organic miscible solvent such as ethanol or acetone in aqueous solution, which, in turn causes a remarkable change in the dielectric constant and solubility parameter of the resulting hydro-organic solvent that eventually leads to the precipitation of the lipid in the new system. This lipid particulate which constitutes the core body of the SLNs becomes limited to the nano-sized via using high shear mixing as well as the presence of stabilizers in the preparation medium [22]. The simplicity and possibility of avoidance of toxic solvents in the SLNs preparation process described earlier are the main advantages of this method. Ethanol is highly soluble in water with minimal interfacial tension, so it diffuses very quickly in aqueous phase thereby resulting in the precipitating of lipids which in the presence of a stabilizer leads to the formation of nanoparticles. In this study some modifications were applied on the characteristics of standard solvent diffusion method, where addition of ethanolic solution to the aqueous phase was performed at about 50 °C. In fact it was seen that if the temperature was upper than the melting point of the lipids, the smaller and more stable particles were produced. It may be due to more rapid evaporation of organic phase (ethanol) which causes lower tendency of lipid phase to aqueous phase. This property possibly forms smaller particles. In addition high temperatures yield more time for surfactants to adsorb on lipid particles and stabilize them.[8] In preliminary tests through using experimental design approach, it was determined that surfactant concentrations affect the production of SLNs, with their particle size being considered as the response. Other conditions such as stirring rate (in a domain of 500–2000 g), rate of ethanolic phase addition to aqueous phase and etc., have no considerable effect on the particle size as response. Hence these conditions were kept constant. Such approach for comparison of different surfactants effect has been observed in several studies [8, 10, 15, 17]. This approach will help to evaluate the effect of surfactant only and without any other interactions. The lipids, repaglinide and surfactants (except PVA which dissolves in water phase) were completely soluble in ethanol. Among the different surfactants used, the phosphatidylcholin-based SLNs showed most opalescence characteristics and the tween 80-based formulations showed least one. The PVP-based SLNs showed lowest yield of nanoparticle production, which can be attributed to its low emulsifying capability.

### Effect on the particle size and zeta potential of SLNs

The particle size and zeta potential of different formulations are shown in Table 2. It has been showed that particles with submicron sizes, specially smaller than 400 nm, could improve bioavailability of drugs [23, 24] and in our study, as indicated, all the SLNs samples showed submicron sizes in a desirable range (83–326 nm). So these nanoparticles are favorable in oral drug delivery systems. However the extent of bioavailability may be dependent to nanoparticles size and usually it is increased by decreasing the size [23, 25]. Hence bioavailability measurement is useful to get a more complete perception of results of this study and it should be investigated later. It had been observed that low molecular surfactants form smaller particles [16]. The reason is their ability to cover interfacial surfaces more quickly than high molecular surfactants [10]. The results of this study agreed those observations, where tween 80, pluronic and lecithin based SLNs formed smaller particle sizes than PVA and PVP based SLNs. Schubert et al. reported that at least 10 % lecithin is necessary to form nanoparticles [18]. However in this study SLNs formed using lower percents of lecithin. The presence of a non-ionic hydrophilic surfactant like tween 80 seems to lead to smaller particles while the phospholipid and co-polymer-based stabilizers made intermediate sizes and the poly-vinyl-based stabilizers led to largest particles among others. The lowest size was observed when a mixture of tween 80 and phosphatidylcholin was used. Blending of surfactants can decrease the particle size and this property was observed in several investigations [12, 16, 18–20, 26, 27]. The results of our study confirm that theory, while addition of tween 80 to phosphatidylcholin and pluronic f127 decreases the particle sizes. The zeta potentials of all formulations were in the negative region, which related to the negative charge of lipids, however, the absolute value of this parameter was dependent to the applied surfactant. This feature of SLNs has been confirmed by various studies [19, 20, 26]. Phosphatidylcholin and mixtures containing it, due to its ionic characteristic, showed the highest surface potential. In fact phosphatidylcholin due to its ionic structure forms an electric double layer on the surface of nanoparticles and this layer increases the zeta potential [10]. Nonionic surfactants stabilize SLNs through their steric properties [19]. The mixture of ionic surfactant like phosphatidylcholin and a nonionic surfactant like pluronic f127 forms more stabilized SLNs [12, 18, 19]. It seems that the molecular weight, structure and conformation play an important role on the quantity of zeta potential of SLNs. Presence of relatively high zeta potential value led to more stable nanoparticles and overcomes the tendency of aggregation due to wander walls attraction forces [10].

**Table 2:** The particle size, zeta potential and loading parameters of different SLNs

Stabilizer(s) used	Size (nm)	Zeta Potential (mV)	Entrapment Efficiency (percent)	Loading Efficiency (percent)
Phosphatidylcholin	203 ± 7	(−)54 ± 8	85 ± 4	7.1 ± 2
Phosphatidylcholin + tween80	91 ± 4	(−)29 ± 4	35 ± 2	4.7 ± 2
Phosphatidylcholin + Pluronic	169 ± 11	(−)46 ± 9	83 ± 4	5.4 ± 1
Pluronic	210 ± 15	(−)43 ± 8	78 ± 5	6 ± 3
PVP	326 ± 24	(−)27 ± 5	28 ± 2	2.8 ± 1
PVA	265 ± 14	(−)26 ± 7	57 ± 3	4.5 ± 2
Tween 80	83 ± 3	(−)22 ± 3	27 ± 3	3.1 ± 1

### Drug loading parameters

The drug loading efficiency and entrapment efficiency of different formulations have been shown in Table 2. The majority of the formulations, as shown, had remarkable drug entrapment efficiency as well as drug loading. The drug solubility in matrix solvent, miscibility in lipid phase and the lipid phase polymorphic state have shown to affect the loading quantity [4, 6, 28, 29]. Repaglinide is poorly soluble in water [30], so it causes low tendency to escape from lipid carrier and thus as indicated, generally it shows high entrapment efficiency. Stearic acid forms lipid structures with high crystalinity [31]. This polymorphic state repels the drug from SLN core to its surface and to the matrix of mixture [28]. GMS was added to lipid phase to decrease the degree of lipid phase crystalinity and to increase loading capacity. As indicated, the loading parameters show an increase by ascending the particle size from the smallest sizes (83 nm, 27 %) until medium sizes (203 nm, 85 %). More increase in the size of particles showed a decrease in loading parameters. In some previous studies, the reverse relation between particle size and loading parameters has been showed [18]. Lowering the particle size increases the specific surface area and thus increase drug loading. The latter part of our finding can be explained by this theory. However since limited number of surfactants were used by Schubert et al., extension of this theory in all conditions such as lower particle sizes in our study, is not possible. Mehnert et al., mentioned mobility of drug after incorporation in SLN through particle surfaces [16]. It is obvious that the bigger surface leads to a higher mobility and tendency of drug to escape from the carrier. So it may be concluded that in smaller particles like tween 80-based SLNs, the whole particle surface is larger and drug repulsion is higher. Therefore loading parameters in these smaller particles are lower relatively. After evaluation of the size and drug loading parameters, the phosphatidylcholin/pluronic-based SLNs which showed maximum loading efficiency (85 %) with eligible particle size (203 nm) was selected for further evaluation.

### Morphological characterization of SLNs

**Fig. 1:**
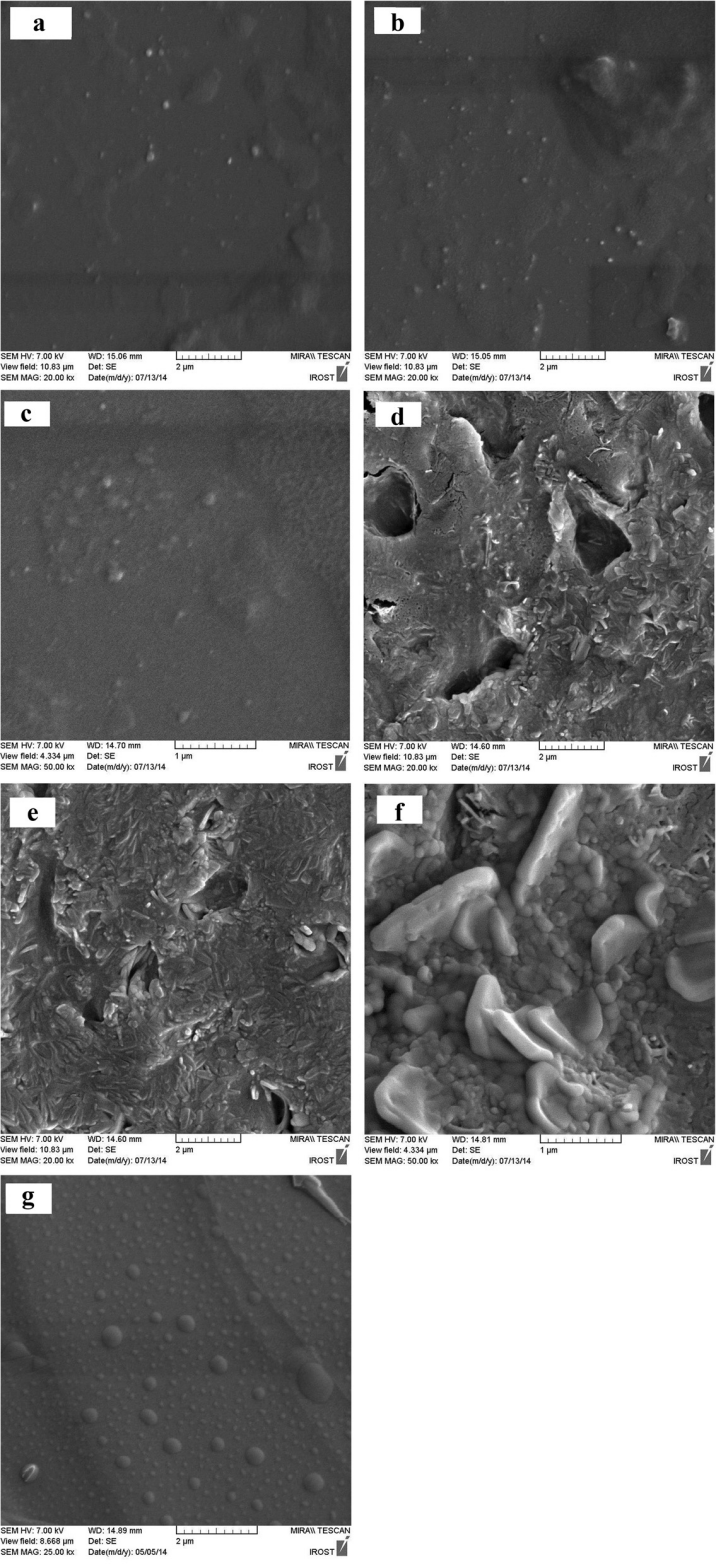
SEM images of different formulations: **a**: tween 80, **b**: tween 80 + phosphatidylcholin, **c**: phosphatidylcholin, **d**: PVA, **e**: PVP, **f**: pluronic F127, **g**: phosphatidylcholin + pluronic F127

SEM images of the nanoparticles prepared using different stabilizers are shown in Fig. 1. The nanoscale sizes of particles in all formulations are evident, though the shapes and assemblies are different among the nanoformulations. The shapes of all nanoparticles except PVP and PVA-based SLNs were spherical and in these two cases, the particles were in rod forms. The surface of phosphatidylcholin and phosphatidylcholin/pluronic-based NPs are smooth but for others are irregular.

### FTIR spectroscopy analysis

**Fig. 2:**
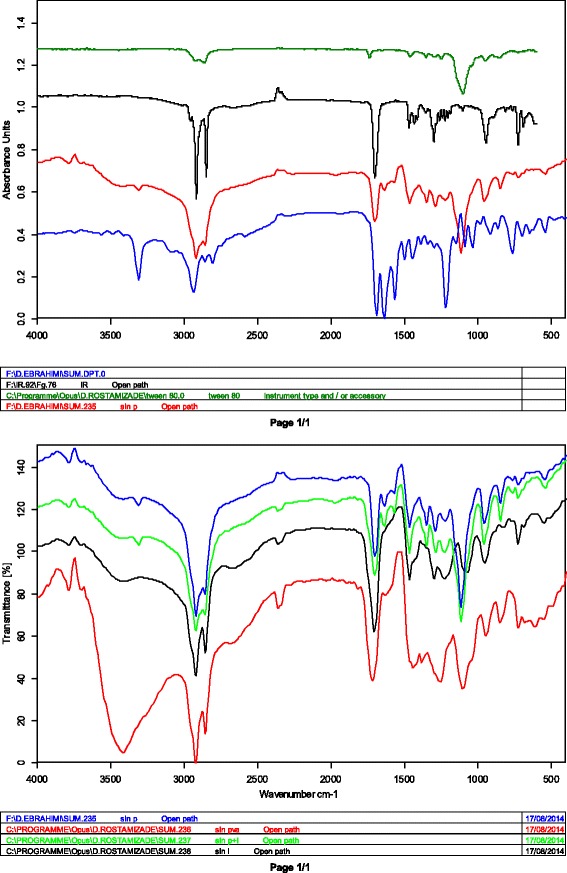
FTIR spectrums of **a**: SLNs-tween80, **b**: stearic acid, **c**: SLNs-PVP, **d**: repaglinide, **e**: SLNs-pluronic, **f**: SLNs-PVA, **g**: SLNs-pluronic/phosphatidylcholin, **h**: SLNs-phosphatidylcholin

FTIR spectra of repaglinide, stearic acid and solid lipid nanoparticles are shown in Fig. 2.

FTIR of pure repaglinide showed peaks at 3306 cm − 1 (NH stretching), 2938 cm − 1 (CH stretching), and 1684 cm − 1 (C = O stretching). The bands at 1040 cm − 1 and 1209 cm − 1 are related to C–O stretching in phenylalkylether structure. The bands at 1566 cm − 1 and 1638 cm − 1 are due to aromatic C = C and N–H bending respectively.

The FTIR spectrum of stearic acid showed a strong peak at 1702 cm − 1 (C = O stretching), 2918 cm − 1 (OH stretching) and 2849 cm − 1 (CH stretching). The long-chain bond is seen in 720 cm − 1 and the CH_2_ and CH_3_ bands are seen in around 1550 cm − 1 and 1300 cm − 1 respectively.

The FTIR spectrum of SLNs shows approximately all absorbances of stearic acid with some minor displacements for 2918 cm − 1 (OH stretching) and 2849 cm − 1 (CH stretching) which is related to inter-molecular forces and re-arrangement of molecules in SLNs structure. Additional peaks in SLNs spectrum are probably related to the applied surfactants. Therefore, these data indicate no formation of chemical bonds between the nanocarrier and the drug which, in turn, ensures the stability of the drug main structure during the preparation process.

### Differential scanning calorimetry (DSC) analysis

**Fig. 3:**
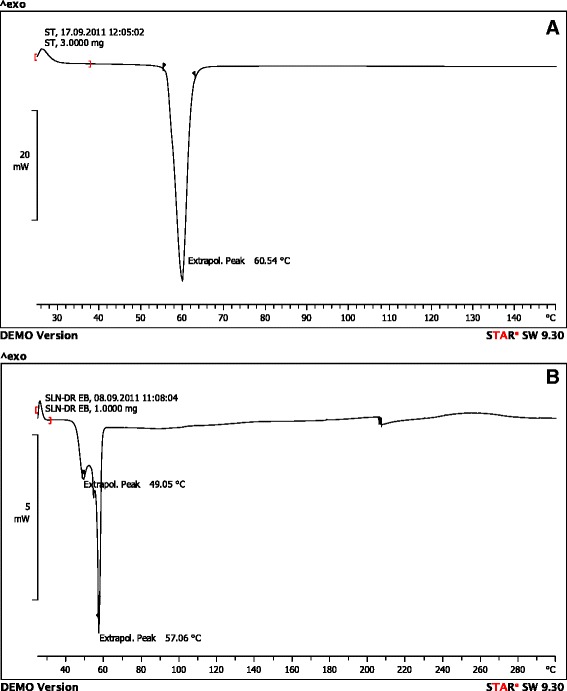
DSC thermograms of **a** = stearic acid, **b** = SLNs

DSC thermograms of stearic acid and SLNs have been shown in Fig. 3. As shown, the peak related to stearic acid melting displaced slightly from 60 °C to 57 °C. The GMS melting point is similar to stearic acid and thus maybe shows interaction with it in this area. The peak related to pluronic F127 is seen lower than 50 °C. All these findings indicate minor displacements of the ingredient peaks which imply new molecular arrangements rather than the formation new chemical bonds between ingredients, a finding supported by the FTIR data.

### *In vitro* drug release

**Fig. 4:**
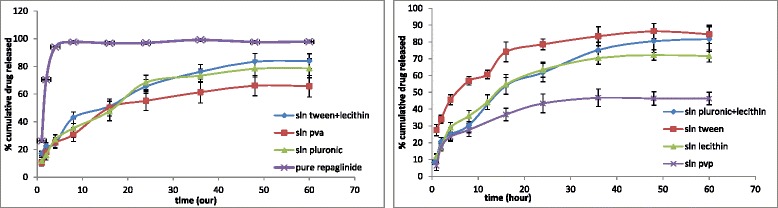
Release data of different SLNs formulations

The dialysis bag method was used to determine the release profile of the drug from SLNs. This is an efficient method to study the drug release from nanocarriers [32]. The release profiles of the drug from seven different SLNs have been shown in Fig. 4. There are clear differences among profiles which have been described by evaluating key factors of the profiles. These key factors are: i) the burst release, ii) the release rate and iii) the maximum drug depletion quantity (MDDQ) (Table 3). The amount of burst release of the drug (total cumulative of drug released in first one hour) from different formulations was significantly different and accordingly, the formulations can be classified into three groups of: low burst release (PVP = 6.4 %, pluronic F127 = 11.6 %, PVA = 10.5 %, phosphatidylcholin/pluronic F127 = 8.4 %, phosphatidylcholin = 11.8 %), medium burst release (phosphatidylcholin/tween 80 = 17.13 %) and high burst release (tween 80 = 27.54 %) (Table 3). The quantity of burst release has direct relation with the amount of drug existed on the surface of nanoparticles [16, 28]. So it can be concluded that the greater the burst release, greater the drug on the particles surfaces. To explain these differences in multiple SLNs, it seems they related to variations in particle sizes since in constant mass of nanoparticles, the smaller ones contain greater particle surfaces and in our study the smaller SLNs showed greater burst release.

**Table 3:** Particle size and release profile parameters of different surfactant formulations

Surfactant/stabilizer	Tween 80	Tween 80+ phosphatidylcholin	Pluronic F127+ phosphatidylcholin	Phosphatidylcholin	Pluronic F127	PVA	PVP
Particle size (nm)	83	91	169	203	210	265	306
Burst release (%)	27.5	17.13	8.4	11.8	11.6	10.5	6.4
Release rate (%/h)	1.49	1.55	1.49	1.63	1.4	1.16	1.12
Max. drug depletion (%)	86.42	84.14	81.68	72.21	78.44	66.14	46.8

The second specification of release profiles is the rate of release. For this purpose, the rate of drug release from the starting point of the ascending part of the profile to reach the plateau was calculated. As it was shown in Table 3, there is no meaningful difference between these formulations in terms of the release rate.

The third factor was the maximum drug depletion quantity (MDDQ). The SLNs due to their condensed and highly ordered molecular conformation, usually retain a part of the loaded drug inside themselves [28]. As it is shown in Table 3, the amount of drug retained in SLNs differs in various formulations significantly.

**Fig. 5:**
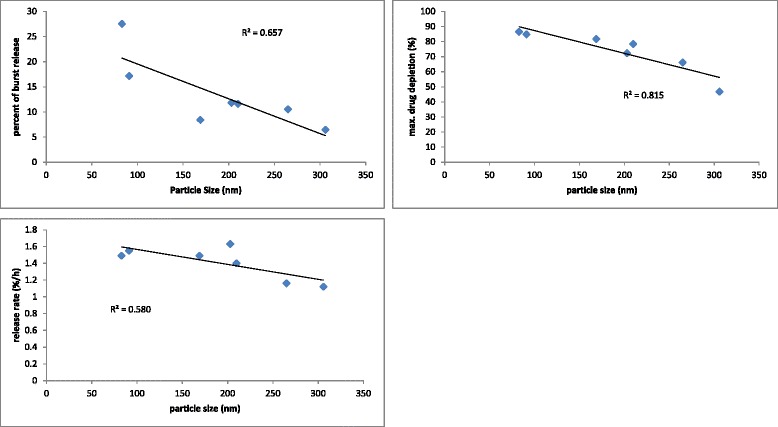
Correlation between particle size and release parameters; burst release, maximum drug depletion and release rate

In order to explore these differences, a correlation analysis was performed between these parameters and their corresponding particle sizes. As indicated in Fig. 5, there is a significant reverse correlation between particle size and the reverse amount of maximum drug depletion quantity (*r*^*2*^ = 0.815). This quantity parameter equals to 0.657 and 0.580 for the values of burst release and release rate, respectively.

In explanation of these correlations, it seems that the higher particle sizes of PVP-based SLNs make a bigger total internal lipophilic space for the highly lipophilic drug to be accommodated which becomes smaller and smaller with decreasing particle sizes for other formulations. The higher the ratio of the internal spaces-to-interfaces, the higher the total drug retention in particles would occur. So the lipid core could detain more drugs and thus the MDDQ becomes greater. The other two parameters (burst release and release rate) showed no significant correlation with particle size, as indicated.

### Drug release kinetic study

The results from substitution of the empirical data on different statistical models are shown in Tables 4 and 5. As indicated, the r^2^ and MRE values of the weibull and logarithmic Wagner models showed most fitness with experimental data in most formulations. It means that there are surface and size variations in particles along release process [33, 34].

**Table 4:** R-squared from fitting release data of different formulations on various models

	Phosphatidylcholin	Phosphatidylcholin/pluronic	Pluronic	PVA	PVP	Tween	Tween/phosphatidylcholin
Zero	0.78	0.88	0.85	0.82	0.72	0.73	0.89
First	0.86	0.96	0.92	0.89	0.77	0.85	0.97
Higuchi	0.93	0.98	0.96	0.94	0.88	0.89	0.97
Peppas	0.99	0.97	0.98	0.98	0.98	0.996	0.98
Hixon-crowell	0.84	0.94	0.9	0.87	0.75	0.81	0.95
Square root	0.83	0.93	0.89	0.86	0.75	0.79	0.94
weibull	0.98	0.99	0.99	0.98	0.9	0.98	0.98
Linear Wagner	0.76	0.86	0.84	0.78	0.63	0.77	0.9
Log Wagner	0.98	0.97	0.98	0.99	0.94	0.98	0.96

**Table 5:** The mean relative error (MRE) from empirical data to calculated data from models

	Phosphatidylcholin	Phosphatidylcholin/pluronic	Pluronic	PVA	PVP	Tween	Tween/phosphatidylchTolin
Zero	21.11	22.61	27.14	26.36	37.06	13.24	20.75
First	25.97	21.93	20.31	24.31	35.88	15.14	11.9
Higuchi	14.8	11.28	10.64	12.44	23.75	10.72	7.62
Peppas	4.49	8.49	5.03	7.63	5.03	1.53	7.8
Hixon-crowell	26.83	25.88	22.91	24.78	36.24	16.19	15.44
Square root	27.5	27.4	24.03	24.99	36.44	16.69	16.92
Weibull	4.09	5.12	5.55	6.4	16.74	2.13	7.33
Linear Wagner	42.85	41.13	25.52	25.5	36.89	36.96	18.05
Log Wagner	3.9	8.6	8.16	4.62	13.44	3.29	11.81

In addition the Peppas model showed high fitness with these data which indicates that the diffusion, erosion or relaxation processes probably contribute in release process [35, 36].

To determine the share of each parameter, the release exponent, n, in Peppas equation was calculated and due to its number in different formulations (Table 6), it can be argued that in tween-based and tween/phosphatidylcholin-based SLNs which *n* < 0.43, the mechanism of drug release is fickian diffusion, but in other formulations where 0.51 < *n* < 0.54, the mechanism of release is a combination of fickian diffusion and non-fickian release mechanisms such as erosion and relaxation [35, 36].

**Table 6:** The “n” parameter from peppas equation in different formulations

	Phosphatidylcholin	Phosphatidylcholin/pluronic	Pluronic	PVA	PVP	Tween	Tween/phosphatidylcholin
**n**	0.54	0.52	0.52	0.51	0.52	0.36	0.41

## Conclusion

The results of this study confirmed the effect of surfactant type on SLNs physicochemical properties such as morphological features, loading parameters, particle sizes and drug release kinetic. These variations due to surfactant effect have been noted in previous studies. In addition, an appropriate mixture of surfactants such as phosphatidylcholin/pluronic can modify the properties of repaglinide loaded SLNs so that convert it as a suitable candidate for delivery of repaglinide for specific purposes, e.g., improvement of the drug bioavailability.
